# Birth Defects among Newborns in a Tertiary Care Centre: A Descriptive Cross-sectional Study

**DOI:** 10.31729/jnma.6610

**Published:** 2022-07-31

**Authors:** Madan Khadka, Jyoti Agarwal, Ramesh Shrestha, Dipti Das

**Affiliations:** 1 Department of Obstetrics and Gynaecology, B.P. Koirala Institute of Health Sciences, Dharan, Sunsari, Nepal; 2 Department of Paediatrics, B.P. Koirala Institute of Health Sciences, Dharan, Sunsari, Nepal

**Keywords:** *birth defect*, *congenital malformation*, *prevalence*

## Abstract

**Introduction:**

The incidence of birth defects is 2-3% in the genera! population but it is increasing. An estimated 303,000 newborns die within 4 weeks of birth every year, worldwide, due to congenital anomalies. The objective of this study was to find out the prevalence of birth defects among newborns in a tertiary care centre.

**Methods:**

A descriptive cross-sectional study was done in the Department of Obstetrics and the Gynaecology and Department of Paediatrics from 15 June 2016 and 14 June 2019. Ethical approval was obtained from the Institutional Review Committee (Reference number: 142/077/078-IRC). Data of newborns were collected from the hospital records. Convenience sampling method was used. Point estimate and 99% Confidence Interval were calculated.

**Results:**

Among 32,695 newborns, birth defects were seen in 169 (0.51%) (0.41-0.61, 99% Confidence Interval). The most common birth defect was musculoskeletal defects seen in 60 (35.50%) newborns followed by central nervous system defect seen in 30 (17.75%) newborns.

**Conclusions:**

The prevalence of birth defects among newborns was lower than in other studies done in a similar setting.

## INTRODUCTION

Congenital anomalies can be defined as structural or functional anomalies that occur during intrauterine life and can be identified prenatally, at birth, or sometimes may only be detected later in infancy, such as hearing defects.^1^ Around 6% of babies are born with a congenital defect, resulting in hundreds of thousands of deaths worldwide.^1^ The birth defect has caused tremendous economic burdens to both society and families and has reduced the average life expectancy and quality of newborns.^2,3^

The prevalence of birth defects in our region i s significantly under-reported. The prevalence of congenital anomalies requires adequate reporting to identify the burden of disease. Adequate data on prevalence and pattern are required to establish baseline rates, provide ideas on the aetiology and help prevention of preventable birth defects and timely intervention for the termination in the poor foetal outcomes.

The aim of this study was to find out the prevalence of birth defects among newborns in a tertiary care centre.

## METHODS

A descriptive cross-sectional study was conducted among newborns delivered at the Department of Obstetrics and Gynaecology and Department of Paediatrics at B.P. Koirala Institute of Health Sciences. Ethical approval was obtained from the Institutional Review Committee of the same institution (Reference number: 142/077/078-IRC). Data from 15 June 2016 to 14 June 2019 was collected from hospital-based records. Cases were taken from labour room records and case records from the nursery or neonatal intensive care units (NICU) of the paediatric ward in which birth defects were detected after admission or later. All the delivery within the institute during the study period were included in the study. All the defects were detected, either antenatally while doing an anomaly scan after 20 weeks of gestation, immediately after delivery of the baby, or during the postnatal period after evaluation by the paediatrician. The pregnancy with antenatal diagnosis of malformation was admitted and delivered subsequently. Thus, all cases who were detected as malformed during the antenatal, intranatal or postnatal period were enrolled. Children with birth defects who were referred from other hospitals were excluded. Convenience sampling method was used.

The sample size was calculated using the following formula:


$$\begin{array}{ll} \text{n} & ={\text{Z}}^{2}\times \frac{\text{p}\times \text{q}}{{\text{e}}^{\text{2}}}\\ & =2.576^{2}\times \frac{0.50\times 0.50}{0.01^{2}}\\ & =16,564 \end{array}$$

Where,

n = minimum required sample size
Z = 2.576 at 99% Confidence Interval (CI)
p = prevalence of birth defect, 2.39%^4^
q = 1-p
e = margin of error, 1%

After doubling the calculated sample size we have included 32,695 newborns for the study.

Birth defects were classified according to the International Classification of Diseases (ICD) classification of birth defects.^4^

Data were entered and analysed using Microsoft Excel 2013 and IBM SPSS Statistics 16.0. Point estimate and 99% CI were calculated.

## RESULTS

**Figure 1:**
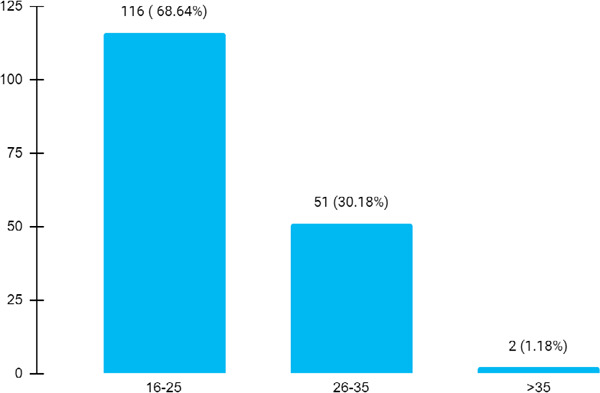
Birth defects according to the age of the mother of the newborns (n= 169).

Among 32,695 newborns, birth defects were seen in 169 (0.51%) (0.41-0.61, 99% CI). Birth defects were found in 116 (68.54%) newborns born to mothers aged 16 to 25 years (Figure 1).

**Figure 2:**
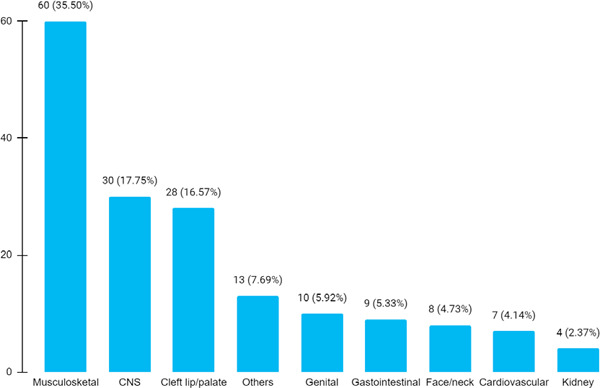
Distribution of birth defects as per ICD classification (n= 169).

The most common birth defect was musculoskeletal defect seen in 60 (35.50%) newborns followed by central nervous system defect in 30 (17.75%) (Figure 2).

**Figure 3:**
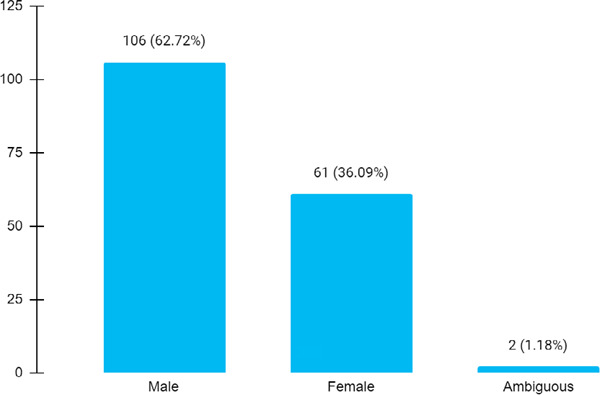
Gender distribution of birth defects (n= 169).

Out of the 169 newborns with birth defects, 106 (62.72%) were males and 2 (1.18%) were ambiguous newborns (Figure 3).

A total of 133 (78.69%) birth defects were detected only after delivery of the newborn. A history of parental consanguinity was present in 3 (1.77%) newborns (Table 1).

**Table 1:** Birth details of the newborns with birth defects (n = 169).

Detection of birth defect	n (%)
After delivery	133 (78.69)
Antenatally using ultrasonography	36 (21.30)
**Timing of delivery**	
Term	133 (78.69)
Preterm	50 (29.58)
Post-term	6 (3.55)
**Birthweight**	
Normal (2.50-3.50 kg)	104 (61.53)
Low (<2.50 kg)	64 (37.86)
**Outcome**	
Live birth	139 (82.24)
Still birth	30 (17.75)

Foetal alcohol syndrome was found in 3 (1.77%) newborns (Table 2).

**Table 2:** Some uncommon birth defects of the study (n = 169).

Birth defects	n (%)
Foetal alcohol syndrome	3 (1.77)
Dandy walker syndrome	2 (1.18)
Down's syndrome	2 (1.18)
Pierre robin sequence	1 (0.59)
Treacher collin syndrome	1 (0.59)
Conjoint twin	1 (0.59)
Ectopia cordis	1 (0.59)
Congenital harlequin baby	1 (0.59)
Suspected congenital cardiac tumour	1 (0.59)

## DISCUSSION

The prevalence of birth defects in our centre was around 50 per 10,000 which was lower than the prevalence in the general population which is 2-3%. A study was done on the prevalence of birth defects in Glasgow, the United Kingdom the Glasgow Register of Congenital anomalies for the period of 1980-1997 found a prevalence of 382 per 10,000.^5^ Congenital heart disease was one of the common birth defects in their study. In a study done in a maternity hospital in Rio de Janeiro, Brazil, the prevalence was 1.7% which was similar to our study. Neural tube defects were the most frequent malformation in their study.^6^ The most frequent birth defect in our study was a musculoskeletal deformity, and club foot/congenital talipes equinovarus (CTEV) were the most common.

The low prevalence of birth defects in our study could be due to parental age where most pregnancies occur between 20-30 years of age. Younger parents (<25 years) are less likely to have a malformed fetus.^7^ Another reason could be the poor detection rate of birth defects during antenatal, intranatal and postnatal periods. Only obvious gross malformation of the newborn were detected while malformations that were not obvious might go unnoticed owing to poor diagnostics tools. None of the stillbirth newborns was autopsied for the cause of the death which could largely miss the malformation in the newborn.

Neural tube defect was the second most common birth defect in our study which is similar to the study done in the United States.^8^ This could be probably due to a lack of folic acid intake and for causal relation, we need further study. Our study showed birth defects predominantly among the male newborns (62.70%) which was in contrast to the study in Kathmandu where the prevalence was 34.60% among males.^9^ However, a study done in the UK on sex prevalence of major congenital anomalies showed a male:female ratio of 1.26,^10^ which was slightly lesser than our study.

In our study, the majority of diagnoses, i.e, 133 (78%) birth defects were detected postpartum thereby emphasising the need for proper diagnostic tools like anomaly scan/targeted sonography in the second trimester, serum analytes like a triple or quadruple marker, amniocentesis need for regular antenatal care (ANC) visits.

In our study, 139 (80%) of all birth defects were live birth while 17.80% were stillbirths with birth defects. Those birth defects that were alive but had poor neonatal outcomes were mostly left neglected by the parents denying further treatment in Nursery and NICU highlighting the poor socioeconomic background. We were unable to follow the outcome of those neonates as those neonates were taken home without further treatment with leave against medical advice. According to European Surveillance of Congenital Anomalies (EUROCAT), 80% of birth defects were live birth similar to our study.^11^

Prevalence of chromosomal anomalies was poorly detected in this study. We recommend future studies with chromosomal study amongst the newborn with suspected chromosomal disorders.

## CONCLUSIONS

The prevalence of birth defects was lower than in the other studies done in a similar setting. Early detection and intervention could have an impact on the prevalence of this condition. This might guide the policymakers to increase the surveillance and raise awareness regarding the impact of the birth defect.
